# Can CRISPR help in the fight against parasitic worms?

**DOI:** 10.7554/eLife.44382

**Published:** 2019-01-31

**Authors:** Paul McVeigh, Aaron G Maule

**Affiliations:** School of Biological SciencesQueen's University of BelfastBelfastUnited Kingdom

**Keywords:** *Schistosoma mansoni*, *Opisthorchis viverrini*, CRISPR/Cas9, gene editing, parasitic worms, cancer, Other

## Abstract

The first reports of CRISPR/Cas9 genome editing in flatworms could usher in a new era of research on these dangerous human parasites.

**Related research article** Ittiprasert W, Mann VH, Karinshak SE, Coghlan A, Rinaldi G, Sankaranarayanan G, Chaidee A, Tanno T, KumKhaek C, Prangtaworn P, Mentink-Kane MM, Cochran CJ, Driguez P, Holroyd N, Tracey A, Rodpai R, Everts B, Hokke CH, Hoffmann KF, Berriman M, Brindley PJ. 2019. Programmed genome editing of the omega-1 ribonuclease of the blood fluke, *Schistosoma mansoni*. *eLife* **8**:e41337. doi: 10.7554/eLife.41337

**Related research article** Arunsan P, Ittiprasert W, Smout MJ, Cochran CJ, Mann VH, Chaiyadet S, Karinshak SE, Sripa B, Young ND, Sotillo J, Loukas A, Brindley PJ, Laha T. 2019. Programmed knockout mutation of liver fluke granulin attenuates virulence of infection-induced hepatobiliary morbidity. *eLife* **8**:e41463. doi: 10.7554/eLife.41463

Parasitic worms, such as roundworms and flatworms, are amongst the most complex pathogens on Earth. They infect people and animals across the globe, and are the sixth leading cause of morbidity worldwide, being responsible for multiple neglected tropical diseases (World Health Organization, 2018; Hotez et al., 2008). There are no vaccines to prevent these infections, and while drug treatments are available, they have a narrow spectrum of action, their efficiency is poor, and there are problems with drug resistance (Hewitson and Maizels, 2014; Geary, 2012). New treatments are urgently needed, but the parasites are so complex that it is challenging to find tools to explore their biology, which hinders the development of drugs and vaccines.

The flatworms *Opisthorchis viverrini* and *Schistosoma mansoni* are two species responsible for human disease. *S. mansoni* causes schistosomiasis, which kills over 200,000 people every year (Lewis and Tucker, 2014). Waterborne *S. mansoni* larvae burrow into human skin and find their way into the bloodstream. Their eggs can become embedded in tissues and release molecules that cause inflammation and chronic disease, such as the omega-1 ribonuclease (coded by the *Sm-omega-1* gene). People get infected with *O. viverrini* when they eat undercooked fish that carry the larvae. The parasites settle into the liver, where they can trigger cholangiocarcinoma, a type of bile duct cancer that has one of the highest mortality rates of any cancer (Sripa et al., 2012). An *O. viverrini* protein called granulin, which is coded by the *Ov-grn-1* gene, may encourage liver cells to multiply abnormally.

The past decade has seen a concerted effort to sequence the genomes of parasitic worms, and more than 150 are available on the community-driven website WormBase ParaSite (Howe et al., 2017). Genomic tools that help dissect the roles of the parasites’ genes are now required to effectively exploit these datasets. In some species, RNA interference methods can silence gene transcripts, but these techniques can only knock down a gene — they cannot increase its expression or incorporate a new piece of genetic information. This limits the range of phenotypes that can be obtained by manipulating a given target (Dalzell et al., 2012). Also, while it is possible to introduce new sequences into schistosome genomes, these changes remain transient (Beckmann and Grevelding, 2012).

CRISPR/Cas9 is a new genetic tool that has revolutionized functional genomics in many organisms by triggering precise, heritable changes to a genome. In 2017, a team at UCLA used CRISPR to manipulate the genetic information of human-parasitic roundworms (Gang et al., 2017). Now, in two papers in eLife, researchers report having harnessed CRISPR/Cas9 to edit the genomes of *O. viverrini* and *S. mansoni* flatworms (Arunsan et al., 2019; Ittiprasert et al., 2019).

From a technical perspective, CRISPR methods require several components to get inside the target cell or tissue. These elements include an enzyme (often Cas9) that can cut DNA, and a single-guide RNA that helps bring Cas9 to the right cleavage site. There, the enzyme snips both strands of DNA, creating a break that can be repaired through a non-homologous end joining mechanism. This error-prone process can introduce mutations in the target site, potentially deactivating a gene. If desired, CRISPR/Cas9 can also be used to introduce new DNA. In this case, a DNA template is provided alongside Cas9 and the single-guide RNA. The sequence is formatted so that it can be inserted into the cleavage site using homology-directed repair mechanisms.

The two latest papers demonstrate that both non-homologous end joining and homology-directed repair appear active in flatworms. In one paper, Paul Brindley of George Washington University, Thewarach Laha of Khon Kaen University and colleagues – including Patpicha Arunsan, Wannaporn Ittiprasert and Michael Smout as joint first authors – describe how they used electroporation to introduce Cas9 and a single guide RNA into *O. viverrini* cells. The *Ov-grn-1* gene was targeted, and edited through non-homologous end joining (Figure 1A; Arunsan et al., 2019).

**Figure 1.**
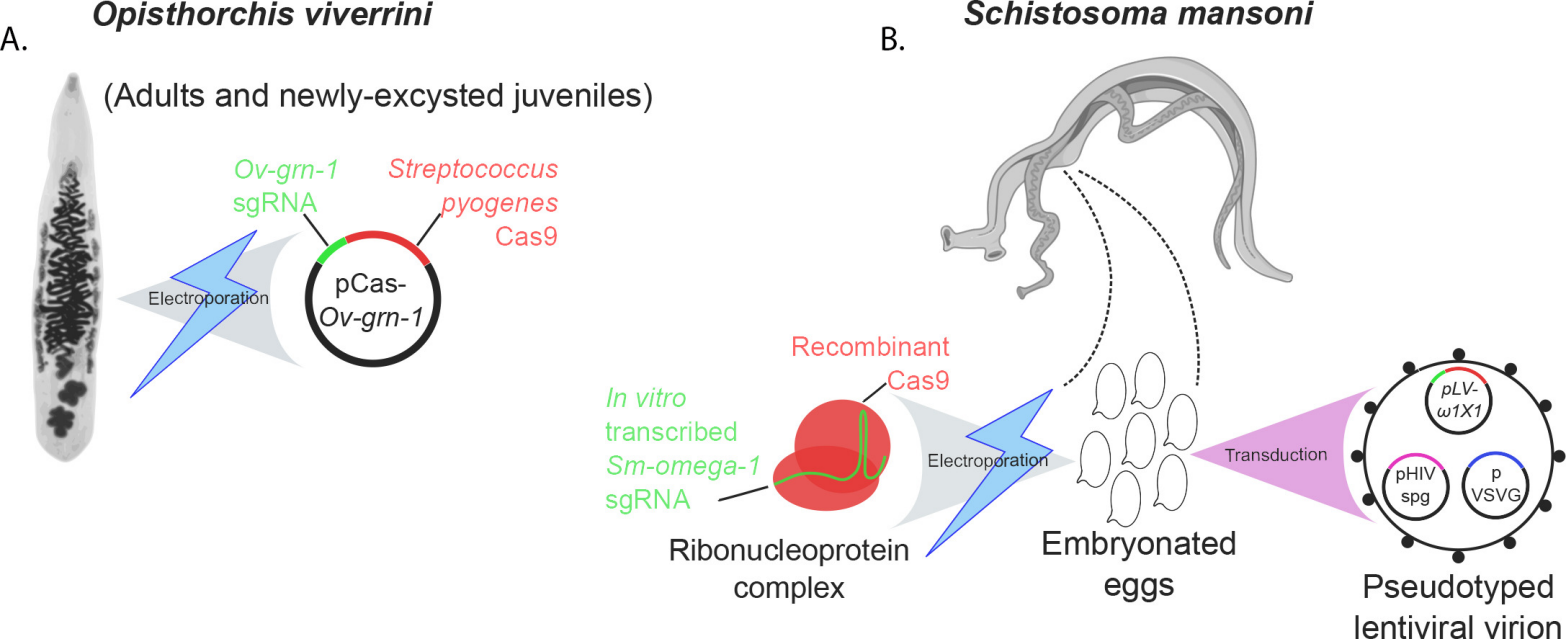
Transfection methods used to generate gene-edited flatworm parasites. (**A**) CRISPR-Cas9 editing of the parasitic flatworm *Opisthorchis viverrini* (left) involved using a technique called electroporation. This forced cells to accept a bacterial plasmid construct (black circle) that encodes a Cas9 enzyme (red) and a guide RNA (green) that targets the *Ov-grn-1* gene in the flatworm. The gene codes for a protein that may help trigger liver cancer in infected individuals. (**B**) The adult female *Schistosoma mansoni* (thinner dark gray worm of the pair) produces eggs while embraced by the male worm (broader light gray worm of the pair). Two methods were used to introduce CRISPR-related materials into these eggs. Electroporation helps the pre-complexed Cas9 protein (red) and strand of guide RNA (green) to get inside the eggs. Alternatively, a lentiviral virion (a particle derived from a virus; circle with black spots) can encapsulate and deliver these elements into cells. The virion also carries several plasmids – *pLV-ω1 × 1* (green and red), *pHIV spg* (magenta), *VSVG* (blue) – which help the virus package and insert Cas9 and the single guide RNA inside cells. Both approaches target the *Sm-omega-1* gene, which codes for a protein that may be involved in damaging the organs of people who carry the worm.

In the other paper, Brindley and co-workers at various institutes in the United States, United Kingdom, Thailand and the Netherlands – including Ittiprasert as first author – report using two methods, electroporation and a viral vector, to deliver CRISPR-related materials to *S. mansoni* eggs (Figure 1B; Ittiprasert et al., 2019). These included a DNA template that encoded a string of stop codons, to be incorporated into *Sm-omega-1* through homology-directed repair.

In both studies, interrupting target genes reduced transcription and led to aberrant phenotypes, even though editing efficiency was very low. For example, in *O. viverrini*, CRISPR-induced mutations were found in fewer than 2% of the targeted sites, yet the levels of *Ov-grn-1* transcript and protein were reduced within 48h of transfection. The livers of animals infected with edited parasites were less swollen, and their biliary ducts had less thickening and scarring compared to the organs of individuals carrying normal worms.

In *S. mansoni*, analyses of genetic variations showed that mutations due to non-homologous end joining were present in less than 4.5% of *Sm-omega-1* target sites, and that the template of stop codons had been incorporated only 0.19% of the time. Despite such low editing efficiency, immune cells reacted less to manipulated eggs than to the non-manipulated ones. Mouse tissues that carried these edited eggs also showed reduced signs of inflammation.

According to both papers, an interplay between gene expression patterns and Cas9 penetrance explains how low editing efficacy can lead to seemingly disproportionate changes in phenotype. The next task could be to determine whether it is possible to improve the editing process, and evaluate how this would influence phenotypic outcomes. Alternatively, homology-directed repair mechanism could be used to label genetically manipulated organisms with a marker (e.g., green fluorescent proteins), which would help separate edited and non-edited animals before examining them for changes in phenotype. Overall, this work shows for the first time how to edit flatworm genomes using CRISPR/Cas9. This will likely initiate a step-change in research into these organisms: it may become possible to create genetically modified worm lines in which to study the biology of the parasites, how they cause disease, and ultimately, how they could be controlled.
